# Global, regional and national incidence, mortality and disability‐adjusted life‐years of skin cancers and trend analysis from 1990 to 2019: An analysis of the Global Burden of Disease Study 2019

**DOI:** 10.1002/cam4.4046

**Published:** 2021-06-09

**Authors:** Wei Zhang, Wen Zeng, Aofei Jiang, Zhi He, Xiaoping Shen, Xian Dong, Jianglong Feng, Hongguang Lu

**Affiliations:** ^1^ Department of Dermatology Affiliated Hospital of Guizhou Medical University Guiyang China; ^2^ Department of Immunology Basic Medical School Guizhou Medical University Guiyang China; ^3^ School of Management Dalian Polytechnic University Dalian China; ^4^ Department of Pathology Affiliated Hospital of Guizhou Medical University Guiyang China

**Keywords:** basal‐cell carcinoma, disability‐adjusted life‐years, global burden of disease, incidence, malignant skin melanoma, mortality, skin cancers, squamous‐cell carcinoma

## Abstract

**Background:**

Information about global and local epidemiology and trends of skin cancers is limited, which increases the difficulty of cutaneous cancer control.

**Methods:**

To estimate the global spatial patterns and temporal trends of skin cancer burden. Based on the GBD 2019, we collected and analyzed numbers and age‐standardized rates (ASR) of skin cancer incidence, disability‐adjusted life years (DALYs) and mortality (ASIR, ASDR, and ASMR) in 204 countries from 1990 through 2019 were estimated by age, sex, subtype (malignant skin melanoma [MSM], squamous‐cell carcinoma [SCC], and basal‐cell carcinoma [BCC]), Socio‐demographic Index (SDI), region, and country. Temporal trends in ASR were also analyzed using estimated annual percentage change.

**Results:**

Globally, in 2019, there were 4.0 million BCC, 2.4 million SCC, and 0.3 million MSM. There were approximately 62.8 thousand deaths and 1.7 million DALYs due to MSM, and 56.1 thousand deaths and 1.2 million DALYs were attributed to SCC, respectively. The men had higher ASR of skin cancer burden than women. The age‐specific rates of global skin cancer burden were higher in the older adults, increasing trends observed from 55 years old. Geographically, the numbers and ASR of skin cancers varied greatly across countries, with the largest burden of ASIR in high SDI regions. However, an unexpected increase was observed in some regions from 1990 to 2019, such as East Asia, and Sub‐Saharan Africa. Although there was a slight decrease of the ASMR and ASDR, the global ASIR of MSM dramatically increased, 1990–2019. Also, there was a remarkable increase in ASR of BCC and SCC burden.

**Conclusions:**

Skin cancer remains a major global public health threat. Reducing morbidity and mortality strategies such as primary and secondary prevention should be reconsidered, especially in the most prevalent and unexpected increased regions, especially for those areas with the greatest proportions of their population over age 55.

## Graphical abstract

Main results of this systematic analysis showed that globally, in addition to a slight fall in mortality of melanoma, there was a dramatic increase in skin cancers burden from 1990 to 2019. Geographically, an unexpected increase was observed in some territories, 1990–2019, such as East Asia and Sub‐Saharan Africa, which were usually considered as low incidence areas in the past. Globally, increased efforts are needed in skin cancer prevention, especially, in regions with a high and/or increased burden of cutaneous cancer.

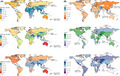

## 1 INTRODUCTION

Skin cancer is one of the most common carcinomas, affecting people of all geographic region, races, and socioeconomic groups.^1^, ^2^, ^3^ It is mainly categorized as MSM and nonmelanoma skin cancers (NMSCs), the latter including BCC and SCC as the major histologic subtypes. Cutaneous carcinoma is becoming a major public health challenge worldwide with a heavy burden of incidence, prevalence, morbidity, and mortality, resulting in substantial economic costs, particularly among Caucasians.^4^ Specifically, NMSCs were the highest incident cancer in 2017 globally.^5^ The epidemiology and burden of skin cancer are associated with demographic trends, socioeconomic development, ethnicity and risk exposures, such as ultraviolet radiation (UVR).^2^, ^6^, ^7^ Early detection and screening of people at high‐risk of skin cancer, the development of new techniques and products for UVR protection, and multiple treatment options have been implemented with the potential to reduce the burden of skin cancer.^8^, ^9^, ^10^ Over the past three decades, however, demographic transformation, socio‐economic development, and risk factors of skin cancer have remarkably changed worldwide.^11^ For instance, a global aging population has rapidly grown due to the decline in fertility and increase in life expectancy.^12^ Stratospheric ozone depletion led to an increase in Ultraviolet‐B (or UVB) component of solar UVR reaching the surface of the Earth.^13^ As a consequence, there was a profound effect on the incidence and burden of skin cancers. Therefore, it is worthwhile to estimate the spatial patterns and temporal trends of skin cancer burden for tailored policy‐making for the global and regional prevention of skin cancer.

Although the trends in the incidence and mortality of skin cancers have been studied in the United States, Australia, and some European countries, and often derive from special surveys or registries of select populations,^14^, ^15^, ^16^, ^17^ there are few publications on skin cancer originating from regions of low incidence or resource‐limited areas of the world. Thus far, accurate global epidemiological information on skin cancers has not been attained. The Global Burden of Diseases (GBD) study fills a gap in which the actual data on disease burden is unavailable or sparse in many countries and territories.^18^ Based on the data of GBD 2019, herein, we estimated the incidence, mortality and DALYs of global skin cancer in 2019 and the temporal trends from 1990 to 2019.

## 2 METHODS

### 2.1 Data source

The burden of disability associated with a disease or disorder can be measured in units called disability‐adjusted life years (DALYs). DALYs represent the total number of years lost to illness, disability, or premature death within a given population. Data on annual incident cases, death numbers, DALYs numbers, and the corresponding age‐standardized incidence rate (ASIR), age‐standardized DALY rate (ASDR) and age‐standardized mortality rate (ASMR) of skin cancer from 1990 to 2019 were collected at the global, regional, and national levels, by age, sex, and subtypes via the Global Health Data Exchange (GHDx) query tool (http://ghdx.healthdata.org/gbd‐results‐tool). Geographically, the world was classified into 21 regions. Moreover, 204 countries and territories were grouped into five Socio‐demographic Index (SDI) groups, including low, low‐middle, middle, high‐middle, and high SDI. The general methods for the GBD 2017 have been detailed in previous studies.^5^, ^18^, ^19^ In brief, skin cancer was defined according to the International Classification of Diseases (ICD‐10: C43‐C43.9, D03‐D03.9, D22‐D23.9, D48.5, and ICD‐9: 172–172.9 for MSM; ICD‐10: C44‐C44.9, D04‐D04.9, D49.2, and ICD‐9: 173–173.9, 222.4, 232–232.9, 238.2 for NMSCs), and the GBD 2017 categorized them into two cancer groups: MSM and NMSCs (composed of BCC and SCC).^5^, ^18^, ^20^ The Cause of Death Ensemble Model (CODEm) was used to generate cause‐specific mortality and years of life lost (YLLs) estimates, and DisMod‐MR 2.1 was used to estimate disease burden (incidence, and years lived with disability [YLDs]). All data above and the corresponding 95% uncertainty intervals (UIs) were estimated based on database of cancer registries, the published literature, surveillance data, census data, and others data sources, by location, sex, age group.^5^, ^18^, ^19^ DALYs were calculated as the sum of cause‐specific mortality, YLLs and YLDs.^21^

### 2.2 Statistical analyses

We chose the estimated annual percentage change (EAPC) in ASIR, ASMR, and ASDR to quantify the temporal trends of skin cancer burden worldwide, from 1990 to 2019. The detailed methods of EAPC have been previously reported.^19^, ^22^, ^23^ Briefly, a regression line model was applied to describe the annual percentage changes in ASR, fitting the natural logarithm of the rates i.e., *y* = *α *+* βx *+* ɛ*, where *y *= ln(ASR), and *x *= calendar year. The EAPC in ASR was estimated as 100 × (exp(*β*)‐1), and captured 95% confidence interval (CI).^23^ All statistics were analyzed via R program (R core team version 3.5.3, Vienna, Austria). A two‐sided *p* value <0.05 was regarded as statistically significant.

## 3 RESULTS

### 3.1 Global incidence, mortality and DALYs of skin cancers in 2019

**FIGURE 1:**
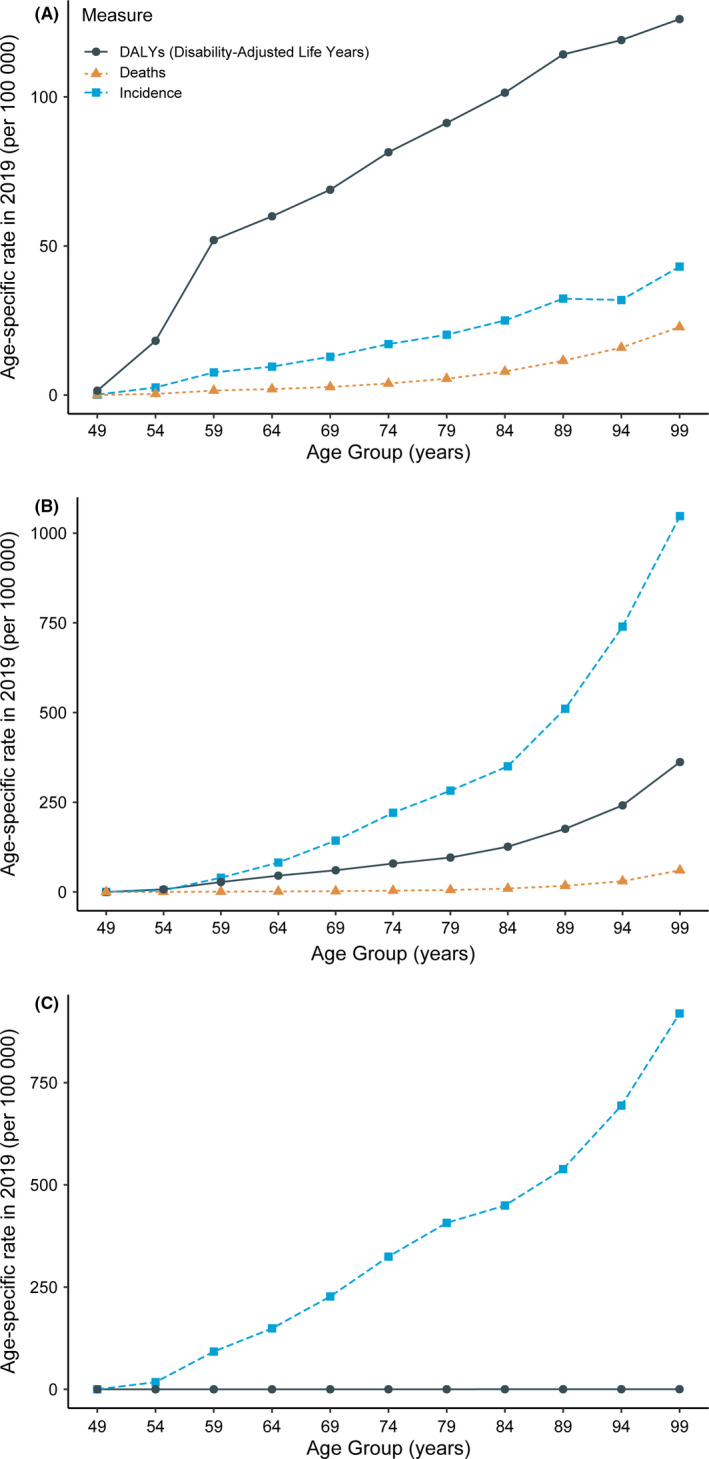
Age‐specific rates of global incidence, disability‐adjusted life years (DALYs), and mortality of skin cancers in 2019. (A) malignant skin melanoma; (B) squamous‐cell carcinoma; (C) basal‐cell carcinoma

Globally, in 2019, for all ages and both sexes combined, the sheer numbers of three incident skin cancers were 4.0 million (95% UI 3.5 to 4.5) BCC, 2.4 million (95% UI 2.1 to 2.7) SCC, and 0.3 million (95% UI 0.2 to 0.3) MSM. There were approximately 62.8 thousand (95% UI 46.3 to 71.0) deaths and 1.7 million (95% UI 1.3 to 2.0) DALYs were due to MSM, and 56.1 thousand (95% UI 50.4 to 59.8) deaths and 1.2 million (95% UI 1.1 to 1.3) DALYs were attributed to SCC. The numbers and ASRs in incidence, mortality, and burden of three skin cancers in men were higher than those in women (Table 1). Figure 1 showed age‐specific rates of global burden of skin cancers in 2019. The rates of global skin cancer burden were higher in the older adults, increasing trends observed from 55 years old in all the subtypes.

**TABLE 1:** Incidence, disability‐adjusted life years (DALYs), and mortality of global skin cancers in 1990 and 2019 and the temporal trends from 1990 to 2019

Characteristics	1990						2019						EAPC (1990–2019)		
	Incidence		DALYs		Death		Incidence		DALYs		Death		ASIR	ASDR	ASMR
	Numbers (95% UI)	ASR No. ×10^−5^ (95% UI)	Numbers (95% UI)	ASR No. ×10^−5^ (95% UI)	Numbers (95% UI)	ASR No. ×10^−5^ (95% UI)	Numbers (95% UI)	ASR No. ×10^−5^ (95% UI)	Numbers (95% UI)	ASR No. ×10^−5^ (95% UI)	Numbers (95% UI)	ASR No. ×10^−5^ (95% UI)	(95% CI)	(95% CI)	(95% CI)
Malignant skin melanoma															
Global															
Both	107380 (85128–134056)	2.56 (2.05–3.25)	1025669 (852906–1299132)	23.58 (19.62–29.92)	33083 (27827–43094)	0.85 (0.72–1.10)	289953 (214481–341965)	3.56 (2.63–4.19)	1707846 (1295585–1997465)	20.81 (15.78–24.33)	62844 (46317–71001)	0.79 (0.58–0.89)	1.13 (0.93–1.32)	−0.49 (−0.57, −0.41)	−0.27 (−0.36, −0.19)
Male	52168 (34828–70914)	2.71 (1.84–3.77)	559273 (404805–750910)	26.88 (19.37–36.32)	17624 (12796–24154)	0.99 (0.73–1.39)	153084 (89756–193326)	4.05 (2.34–5.06)	973502 (642004–1210394)	24.83 (16.21–30.76)	35425 (21971–42688)	0.98 (0.60–1.17)	1.38 (1.16–1.59)	−0.33 (−0.42, −0.24)	−0.04 (−0.14, 0.05)
Female	55212 (41148–72928)	2.48 (1.85–3.29)	466397 (392491–645042)	20.68 (17.43–28.75)	15459 (12944–21686)	0.73 (0.61–1.03)	136869 (92692–166593)	3.20 (2.18–3.90)	734345 (524702–882039)	17.28 (12.43–20.80)	27418 (18967–31888)	0.63 (0.44–0.74)	0.87 (0.69–1.05)	−0.68 (−0.75, −0.61)	−0.53 (−0.61, −0.46)
Socio‐demographic Index															
High	76420 (57943–95320)	7.86 (5.93–9.66)	488331 (364154–610094)	50.60 (37.73–62.56)	16536 (12750–21901)	1.64 (1.26–2.16)	194489 (139700–237650)	12.40 (9.18–15.50)	743189 (521411–906594)	48.06 (34.82–59.93)	29419 (19479–34234)	1.63 (1.12–1.93)	1.79 (1.57–2.02)	−0.22 (−0.28, −0.16)	−0.04 (−0.09, 0.01)
High‐middle	22402 (18294–20247)	2.04 (1.68–2.72)	302047 (252900–391620)	26.97 (22.65–35.02)	9746 (8319–13057)	0.93 (0.80–1.25)	67629 (47547–78643)	3.54 (2.48–4.10)	490829 (356720–558876)	25.70 (18.81–29.57)	17965 (12942–20247)	0.91 (0.66–1.03)	2.02 (1.85 –2.19)	−0.33 (−0.49, −0.17)	−0.15 (−0.31, −0.01)
Middle	4988 (3866–6581)	0.44 (0.35–0.58)	126906 (96412–173680)	10.04 (7.69–13.55)	3770 (2905–5046)	0.37 (0.29–0.48)	17303 (13663–20507)	0.70 (0.55–0.83)	243560 (191742–291014)	9.51 (7.54–11.33)	8559 (6747–10203)	0.37 (0.29–0.44)	1.84 (1.73–1.96)	−0.19 (−0.23, −0.16)	0.00 (−0.04, 0.04)
Low‐middle	2103 (1497–3034)	0.32 (0.23–0.44)	61650 (42187–92963)	8.07 (5.67−11.82)	1768 (1254−2564)	0.30 (0.22−0.41)	6124 (4789−7242)	0.42 (0.33−0.50)	130964 (101688−157188)	8.52 (6.63−10.26)	4158 (3260−5001)	0.31 (0.24−0.37)	1.03 (0.99−1.07)	0.19 (0.17, 0.22)	0.19 (0.16, 0.23)
Low	1428 (924–2250)	0.50 (0.35–0.73)	46297 (28979–77576)	14.18 (9.40–21.77)	1248 (831–1931)	0.48 (0.34–0.69)	3222 (2445–4161)	0.51 (0.38–0.65)	98510 (74008–127212)	13.79 (10.24–17.49)	2713 (2014–3439)	0.47 (0.35–0.59)	0.22 (0.14, 0.30)	−0.13 (−0.15, −0.1)	−0.08 (−0.09, −0.06)
Malignant skin melanoma															
Region															
Asia Pacific, high income	1705 (1466–2237)	0.88 (0.75–1.16)	13346 (11901–18063)	6.78 (6.06–9.16)	458 (402–626)	0.24 (0.21–0.33)	5743 (3920–6910)	1.76 (1.20–2.11)	22747 (15186–26687)	7.07 (4.79–8.27)	1042 (722–1203)	0.24 (0.16–0.27)	2.65 (2.38, 3.93)	0.21 (0.09, 0.33)	0.05 (−0.04, 0.14)
Central Asia	552 (397–652)	1.12 (0.79–1.31)	10368 (7582–12138)	19.93 (14.53–23.16)	352 (249–408)	0.76 (0.53–0.88)	1014 (849–1444)	1.34 (1.13–1.90)	14473 (12176−20500)	15.47 (13.02–21.92)	489 (415–683)	0.73 (0.62–1.00)	−0.05 (−0.70, 0.03)	−1.16 (−1.58, −0.74)	−0.92 (−1.35, −0.48)
East Asia	3864 (2717–5427)	0.40 (0.29–0.56)	99670 (66737–143643)	9.52 (6.50–13.93)	2881 (1995–4217)	0.33 (0.24–0.48)	17349 (10980–21918)	0.91 (0.58–1.14)	157822 (102030–196625)	8.08 (5.27–10.05)	5456 (3576–6815)	0.28 (0.19–0.35)	3.28 (2.97,3.58)	−0.52 (−0.64, −0.40)	−0.39 (−0.50, −0.29)
South Asia	1284(917–1800)	0.21 (0.15–0.28)	38905 (27418–56686)	5.26 (3.80–7.50)	1094 (791–1557)	0.20 (0.15–0.27)	3768 (2617–4571)	0.25 (0.18–0.30)	85960 (60777–103182)	5.38 (3.85–6.50)	2660 (1911–3222)	0.19 (0.14–0.24)	0.64 (0.59, 0.7)	0.05 (0, 0.1)	−0.15 (−0.22, −0.07)
Southeast Asia	745 (582–1146)	0.26 (0.21–0.38)	22654 (17258–35353)	6.90 (5.40–10.48)	658 (521–993)	0.25 (0.20–0.36)	1716 (1359–2389)	0.28 (0.22–0.39)	42051 (33965–58361)	6.39 (5.14–8.83)	1434 (1148–1975)	0.25 (0.20–0.34)	0.23 (0.17, 0.28)	−0.32 (−0.34, −0.29)	−0.05 (−0.09, −0.02)
Australasia	7194 (5608–9420)	31.72 (24.55–41.32)	31241 (23438–41436)	138.36 (104.35–182.31)	1013 (787–1399)	4.41 (3.42–6.06)	18373 (12445–24061)	43.36 (30.23–57.02)	54107 (37983–69260)	128.92 (93.12–169.58)	2098 (1389–2511)	4.37 (2.96–5.41)	0.98 (0.79, 1.18)	−0.25 (−0.35, −0.15)	0.01 (−0.1, 0.12)
Caribbean	209 (181–281)	0.75 (0.65–1.01)	3848 (3202–5503)	13.36 (11.18–18.79)	126 (106–175)	0.48 (0.41–0.66)	566 (447–749)	1.11 (0.87–1.46)	7250 (5736–9690)	14.26 (11.26–19.14)	268 (215–358)	0.52 (0.42–0.69)	1.22 (1.15, 1.28)	0.08 (−0.03, 0.18)	0.15 (0.06, 0.24)
Central Europe	5103 (4301–6768)	3.62 (3.06–4.78)	74977 (62565–98648)	53.32 (44.47–69.79)	2473 (2112–3334)	1.76 (1.50–2.37)	13818 (9945–16809)	7.73 (5.66–9.40)	105197 (74961–127277)	59.98 (43.25–73.01)	4144 (2878–4959)	2.09 (1.47–2.52)	2.76 (2.59, 2.94)	0.40 (0.29, 0.52)	0.66 (0.55, 0.77)
Malignant skin melanoma															
Region															
Eastern Europe	6747 (5609–9823)	2.54 (2.10–3.69)	99568 (79908–138634)	37.75 (30.06–52.09)	3173 (2651–4641)	1.19 (0.99–1.74)	17924 (13058–22508)	5.93 (4.36–7.44)	160338 (120985–193182)	54.83 (41.95–66.45)	5399 (4056–6515)	1.70 (1.28–2.06)	3.21 (2.99, 3.42)	0.99 (0.68, 1.31)	1.01 (0.72, 1.29)
Western Europe	34460 (27189–44244)	7.00 (5.47–8.92)	252769 (195891–336947)	51.79 (39.94–67.67)	8898 (7176–12092)	1.67 (1.33–2.26)	99643 (61381–119771)	14.55 (9.16–17.60)	393137 (237792–443322)	57.40 (35.15–65.13)	15922 (9412–17764)	1.89 (1.13–2.10)	2.58 (2.32, 2.84)	0.36 (0.26, 0.46)	0.48 (0.39, 0.56)
Andean Latin America	218 (166–316)	0.97 (0.75–1.37)	5638 (4218–8513)	22.71 (17.32–32.69)	186 (143–264)	0.89 (0.69–1.24)	786 (565–1104)	1.37 (0.99–1.92)	11979 (8569–17172)	20.56 (14.69–29.23)	475 (339–640)	0.86 (0.61–1.15)	1.41 (1.31, 1.51)	−0.20 (−0.28, 0.11)	0.07 (−0.02, 0.16)
Central Latin America	683 (601–994)	0.72 (0.62–1.03)	14901 (13197–22009)	14.34 (12.63–21.36)	472 (410–695)	0.55 (0.47–0.80)	3459 (2710–4716)	1.43 (1.12–1.95)	44445 (34503–61971)	18.16 (14.10–25.22)	1666 (1275–2249)	0.71 (0.54–0.96)	2.30 (2.18, 2.42)	0.75 (0.69, 0.80)	0.82 (0.76, 0.87)
Southern Latin America	732 (630–1077)	1.58 (1.36–2.32)	13090 (11270–19444)	27.90 (24.02–41.48)	448 (389–671)	0.99 (0.85–1.48)	2606 (1817–3486)	3.31 (2.32–4.44)	27609 (19727–34976)	35.44 (25.61–45.44)	1049 (742–1312)	1.28 (0.91–1.60)	2.33 (2.09, 2.57)	0.59 (0.41, 0.78)	0.71 (0.52, 0.91)
Tropical Latin America	1359 (1067–1816)	1.30 (1.01–1.73)	31294 (24617–43260)	27.87 (21.78–38.32)	929 (724–1271)	0.97 (0.75–1.32)	4921 (3944–6936)	2.02 (1.62–2.84)	70759 (58975–103756)	28.61 (23.88–41.90)	2514 (1971–3549)	1.05 (0.82–1.47)	1.47 (1.27, 1.66)	0 (−0.16, 0.17)	0.23 (0.09, 0.37)
North Africa and Middle East	1094 (639–1564)	0.57 (0.34–0.80)	26035 (14697–38550)	12.14 (6.93–17.66)	796 (463–1140)	0.47 (0.28–0.65)	3885 (2641–4669)	0.83 (0.55–1.00)	47804 (32044–58375)	9.58 (6.38–11.64)	1619 (1057–1957)	0.40 (0.26–0.48)	1.28 (1.20, 1.36)	−0.93 (−0.97, −0.88)	−0.62 (−0.66, −0.58)
North America, high income	39624 (28895–47582)	12.18 (8.81–14.43)	231788 (162648–277367)	72.36 (50.75–86.30)	7588 (5575–9540)	2.25 (1.65–2.81)	90002 (67857–118878)	16.62 (13.09–22.68)	342133 (264823–458947)	64.24 (51.69–89.70)	13202 (9438–16648)	2.20 (1.63–2.85)	1.86 (0.63, 1.09)	−0.55 (−0.61, −0.50)	−0.18 (−0.23, −0.14)
Malignant skin melanoma															
Region															
Oceania	16 (11–27)	0.48 (0.34–0.83)	478 (317–854)	12.49 (8.49–22.01)	14 (10–25)	0.49 (0.34–0.84)	36 (24–57)	0.48 (0.33–0.74)	1073 (694–1756)	12.13 (8.08–19.37)	33 (22–52)	0.48 (0.34–0.75)	−0.01 (−0.04, 0.02)	−0.04 (−0.07, −0.01)	0.02 (−0.01, 0.06)
Central Sub‐Saharan Africa	166 (108–281)	0.64 (0.44–0.93)	5288 (3297–9736)	17.11 (11.51–26.57)	148 (99–231)	0.63 (0.44–0.88)	412 (295–596)	0.67 (0.48–0.92)	11919 (8415–17619)	16.63 (11.84–23.56)	348 (249–493)	0.63 (0.44–0.86)	0.20 (0.13, 0.26)	−0.08 (−0.12, −0.04)	0.04 (0, 0.08)
Eastern Sub‐Saharan Africa	797 (521–1314)	0.83 (0.57–1.25)	26209 (16519–45722)	23.98 (16.12–37.68)	681 (456–1095)	0.77 (0.54–1.16)	1936 (1436–2685)	0.90 (0.66–1.20)	56660 (42429–78441)	23.36 (17.22–31.48)	1504 (1112–2017)	0.78 (0.57–1.03)	0.28 (0.22, 0.33)	−0.08 (−0.12, −0.04)	0.04 (0.01, 0.07)
Southern Sub‐Saharan Africa	400 (307–529)	1.31 (1.00–1.65)	10263 (7922–13964)	30.55 (23.39–39.96)	320 (243–411)	1.13 (0.84–1.42)	951 (638–1176)	1.59 (1.04–1.94)	21071 (14225–26144)	32.67 (21.66–39.86)	705 (458–856)	1.27 (0.80–1.53)	0.72 (0.64, 0.80)	0.34 (0.19, 0.50)	0.49 (0.33, 0.66)
Western Sub‐Saharan Africa	429 (275–608)	0.42 (0.28–0.56)	13340 (8025–20582)	11.26 (7.20–15.57)	375 (243–521)	0.40 (0.27–0.53)	1047 (707–1329)	0.45 (0.31–0.57)	29312 (20108–38381)	10.85 (7.38–13.83)	815 (559–1038)	0.40 (0.27–0.50)	0.34 (0.27, 0.41)	−0.08 (−0.13, −0.03)	0.08 (0.03, 0.12)
Non‐melanoma skin cancer (squamous‐cell carcinoma)															
Global															
Both	756481 (606882–933155)	22.27 (18.04–27.43)	561292 (518444–598784)	14.42 (13.30–15.40)	23222 (21441–24436)	0.69 (0.63–0.73)	2402221 (2122698–2712803)	30.30 (26.89–34.08)	1181530 (1084052–1262186)	14.64 (13.43–15.64)	56054 (50415–59792)	0.73 (0.65–0.78)	1.13 (0.50, 1.77)	0.20 (0.10, 0.30)	0.41 (0.34, 0.49)
Male	407454 (324920–512867)	28.15 (22.69–35.14)	346680 (312729–371315)	19.59 (17.63–21.01)	13696 (12374–14508)	0.95 (0.86–1.01)	1444114 (1263056–1652684)	41.39 (36.28–47.23)	741595 (681105–800732)	20.02 (18.42–21.62)	33244 (30347–35571)	1.01 (0.91–1.08)	1.38 (0.74, 2.03)	0.19 (0.09, 0.30)	0.40 (0.33, 0.48)
Female	349028 (282227–428879)	18.13 (14.67–22.28)	214612 (196484–238679)	10.28 (9.41 −11.38)	9526 (8681–10355)	0.50 (0.45–0.54)	958107 (844943–1080644)	21.84 (19.26–24.62)	439935 (386241–484874)	10.14 (8.91–11.17)	22809 (19350–25231)	0.52 (0.44–0.58)	0.71 (0.10, 1.33)	0.14 (0.03, 0.26)	0.36 (0.26, 0.45)
Non‐melanoma skin cancer (squamous‐cell carcinoma)															
Socio‐demographic Index															
High	706826 (562544–876862)	67.94 (54.74–84.13)	163231 (146023–184354)	16.03 (14.38–18.06)	6199 (5759–6394)	0.61 (0.56–0.63)	2252560 (1993685–2549163)	116.50 (102.73–132.17)	311830 (268493–365959)	16.64 (14.35–19.52)	13002 (11102–13900)	0.61 (0.53–0.64)	1.97 (1.3, 2.65)	0.2 (−0.1, 0.49)	0.20 (0.09, 0.31)
High‐middle	33081 (28641–38439)	3.52 (3.07–4.06)	162610(153561–170464)	15.84 (14.95–16.60)	7641 (7187–7973)	0.87 (0.80–0.91)	84828 (74907–95693)	4.24 (3.76–4.76)	278963 (255989–298819)	14.12 (12.96–15.12)	15469 (13914–16567)	0.80 (0.71–0.86)	0.67 (0.54, 0.8)	−0.36 (−0.44, −0.27)	−0.22 (−0.28, −0.16)
Middle	11348 (9800–13008)	1.34 (1.18–1.53)	151251 (138925–163475)	14.27 (13.13–15.40)	6009 (5529–6491)	0.73 (0.66–0.78)	44094 (37509–51378)	1.95 (1.67–2.24)	371726 (335341–406668)	15.38 (13.80–16.79)	17710 (15838–19361)	0.84 (0.75–0.92)	1.37 (1.29, 1.54)	0.55 (0.41, 0.69)	0.85 (0.69, 1.01)
Low‐middle	4047 (3518–4598)	0.85 (0.75–0.96)	66519 (54875–76684)	11.04 (8.97–12.59)	2657 (2142–3028)	0.57 (0.45–0.65)	14737 (12683–16987)	1.22 (1.06–1.40)	171270 (152501–187298)	12.73 (11.23–13.96)	7865 (6864–8605)	0.69 (0.59–0.76)	1.31 (1.25, 1.36)	0.68 (0.58, 0.78)	0.9 (0.77, 1.04)
Low	1112 (940–1298)	0.57 (0.50–0.66)	17352 (12141–20837)	7.42 (4.98–8.88)	701 (466–838)	0.39 (0.24–0.47)	8330 (5861–11397)	0.61 (0.53–0.70)	46978 (32488–55884)	8.91 (6.00–10.61)	2688 (2296–3113)	0.48 (0.31–0.57)	0.57 (0.47, 0.67)	0.7 (0.66, 0.73)	0.8 (0.75, 0.85)
Region															
Asia Pacific, high income	1355 (1166–1578)	0.78 (0.67–0.91)	14507 (13906–15001)	7.55 (7.19–7.82)	729 (682–760)	0.43 (0.39–0.45)	6864 (5593–8285)	1.21 (0.99–1.44)	26707 (22953–28897)	6.00 (5.37–6.42)	2072 (1656–2306)	0.36 (0.30–0.40)	1.37 (1.19, 1.54)	−0.8 (−0.89, −0.7)	−0.57 (−0.65, −0.49)
Central Asia	896 (761–1040)	2.05 (1.75–2.36)	4567(4013–4956)	9.58 (8.35–10.43)	197 (168–216)	0.47 (0.39–0.52)	1851 (1582–2127)	2.91 (2.52–3.29)	11287 (9763–12527)	15.57 (13.31–17.14)	476 (404–522)	0.83 (0.70–0.91)	1.4 (1.17, 1.64)	2.32 (2.05, 2.6)	2.72 (2.43, 3.01)
East Asia	5723 (4880–6630)	0.80 (0.70–0.92)	133982 (117575–151252)	14.64 (12.90–16.45)	5180 (4578–5805)	0.70 (0.62–0.78)	41696 (35043–49043)	2.08 (1.76–2.42)	332831 (280835–384492)	16.60 (14.11–19.12)	15905 (13480–18220)	0.87 (0.74–0.99)	3.19 (3.01, 3.37)	1.03 (0.76, 1.3)	1.39 (1.09, 1.69)
Non‐melanoma skin cancer (squamous‐cell carcinoma)															
Region															
South Asia	1622 (1356–1912)	0.39 (0.33–0.45)	38950 (26205–47447)	7.13 (4.67–8.68)	1586 (1035–1935)	0.38 (0.24–0.47)	4507 (3817–5306)	0.38 (0.33–0.44)	90033 (69403–107258)	6.53 (5.02–7.76)	4130 (3139–4905)	0.35 (0.27–0.42)	−0.02 (−0.05, 0)	−0.44 (−0.5 −0.37)	−0.43 (−0.52, −0.35)
Southeast Asia	1871 (1657–2091)	0.86 (0.77–0.96)	43607 (37759–50243)	15.74 (13.65–18.02)	1642 (1418–1874)	0.74 (0.63–0.84)	4718 (4160–15333)	0.88 (0.79–0.98)	97002 (83967–109397)	15.66 (13.52–17.62)	4107 (3557–4596)	0.78 (0.67–0.87)	0.10 (0.04, 0.16)	−0.21 (−0.33, −0.1)	−0.05 (−0.17, 0.06)
Australasia	46825 (40005–54307)	202.89 (174.68–233.86)	8221 (8221–9247)	35.72 (32.33–40.23)	324 (301–346)	1.44 (1.32–1.54)	126454 (106605–148627)	249.75 (210.03–294.74)	19247 (16920–22056)	38.68 (34.11–44.15)	927 (804–1011)	1.70 (1.48–1.85)	0.58 (0.43, 0.73)	0.09 (0.01, 0.16)	0.39 (0.28, 0.51)
Caribbean	654 (582–723)	2.66 (2.38–2.94)	5425 (5085–5801)	20.89 (19.52–22.32)	269 (247–287)	1.15 (1.05–1.23)	1541 (1389–1705)	2.97 (2.68–3.29)	13295 (11186–15377)	25.72 (21.65–29.74)	739 (623–853)	1.41 (1.19–1.63)	0.41 (0.34, 0.47)	0.97 (0.85, 1.09)	0.97 (0.84, 1.09)
Central Europe	5194 (4710–5740)	3.87 (3.51–4.27)	31599 (28828–32548)	23.30 (21.10–24.07)	1774 (1602–1845)	1.47 (1.30–1.54)	9875 (8881–10925)	4.52 (4.07–4.99)	35219 (31119–39591)	17.01 (15.00–19.15)	2366 (2056–2657)	1.08 (0.94–1.21)	0.72 (0.65, 0.79)	−1.35 (−1.5, −1.2)	−1.33 (−1.52, −1.13)
Eastern Europe	6067 (5056–7195)	2.40 (2.01–2.80)	40503 (37517–42799)	15.30 (14.18–16.16)	1831 (1712–1961)	0.75 (0.70–0.81)	10101 (8403–12036)	2.93 (2.44–3.48)	55357 (49136–62205)	17.09 (15.12–19.10)	2894 (2573–3233)	0.86 (0.76–0.96)	0.97 (0.84, 1.11)	0.1 (−0.19, 0.4)	0.28 (0.03, 0.53)
Western Europe	32240 (28714–35726)	5.58 (4.99–6.20)	62068 (58376–63868)	11.29 (10.62–11.61)	3590 (3263–3728)	0.64 (0.57–0.66)	65275 (56889–74318)	6.87 (5.93–7.89)	89530 (79999–94768)	9.90 (9.04–10.44)	6563 (5572–7043)	0.60 (0.52–0.64)	0.89 (0.8, 0.99)	−0.24 (−0.38, −0.11)	0.07 (−0.08, 0.22)
Andean Latin America	420 (372–472)	2.23 (2.00–2.51)	3156 (2777–3680)	14.84 (13.10–17.17)	145 (128–165)	0.81 (0.71–0.91)	1436 (1256–1629)	2.65 (2.31–3.00)	10191 (8165–12331)	18.10 (14.53–21.84)	569 (467–677)	1.06 (0.87–1.27)	0.69 (0.61, 0.77)	0.77 (0.63, 0.91)	1.12 (0.96, 1.27)
Non‐melanoma skin cancer (squamous‐cell carcinoma)															
Region															
Central Latin America	3658 (3149–4197)	4.79 (4.14–5.47)	21064 (19848–21785)	24.18 (22.60–25.08)	922 (853–961)	1.28 (1.16–1.34)	11594 (10005–13283)	5.07 (4.39–5.81)	50565 (43604–58328)	21.46 (18.44–24.75)	2757 (2343–3152)	1.23 (1.04–1.40)	0.34 (0.25, 0.43)	−0.53 (−0.58, −0.49)	−0.23 (−0.28, −0.18)
Southern Latin America	2593 (2247–2955)	5.98 (5.20–6.80)	6628 (6167–7213)	14.80 (13.74–16.16)	326 (301–359)	0.80 (0.73–0.88)	8335 (7276–9461)	9.86 (8.61–11.16)	13297 (12036–14079)	16.10 (14.57–17.05)	803 (704–861)	0.94 (0.83–1.01)	1.62 (1.32, 1.93)	0.31 (0.27, 0.35)	0.66 (0.61, 0.71)
Tropical Latin America	2560 (2192–2967)	2.99 (2.57–3.45)	19449 (18621–20268)	20.68 (19.56–21.60)	801 (753–839)	1.06 (0.97–1.12)	12085 (10224–14065)	5.07 (4.29–5.91)	53225 (48153–56188)	22.10 (19.88–23.36)	2805 (2424–3000)	1.22 (1.05–1.31)	1.89 (1.75, 2.03)	0.35 (0.28, 0.42)	0.72 (0.65, 0.79)
North Africa and Middle East	1503 (1300–1718)	1.03 (0.91–1.16)	15483 (13523–17893)	9.11 (7.81–10.58)	667 (563–778)	0.50 (0.41–0.60)	4347 (3782–4943)	1.15 (1.01–1.30)	38526 (34637–43203)	9.08 (8.11–10.14)	1845 (1636–2063)	0.54 (0.47–0.61)	0.45 (0.26, 0.64)	0.01 (−0.04, 0.06)	0.33 (0.20, 0.47)
North America, high income	641232 (500514–807198)	179.63 (141.66–224.05)	94769 (79206–115168)	27.60 (23.24–33.08)	2528 (2335–2609)	0.72 (0.66–0.74)	2085597 (1843524–2366411)	324.18 (285.94–368.52)	193954 (155776–243557)	30.91 (25.05–38.75)	5000 (4332–5316)	0.75 (0.66–0.79)	2.2 (1.46, 2.95)	0.42 (−0.02, 0.87)	0.29 (0.16, 0.43)
Oceania	14 (13–16)	0.67 (0.61–0.74)	390 (481–317)	13.11 (10.78–16.05)	15 (12–18)	0.70 (0.57–0.85)	38 (34–42)	0.71 (0.65–0.78)	1044 (802–1387)	14.56 (11.53–18.65)	40 (32–52)	0.80 (0.65–0.98)	0.24 (0.21, 0.27)	0.41 (0.35, 0.48)	0.50 (0.44, 0.56)
Central Sub‐Saharan Africa	139 (116–165)	0.77 (0.67–0.88)	1875 (1281–2371)	8.55 (5.48–10.93)	73 (47–93)	0.46 (0.27–0.60)	379 (321–444)	0.85 (0.75–0.98)	5711 (3938–7499)	10.62 (6.98–13.89)	227 (149–298)	0.57 (0.36–0.75)	0.47 (0.37, 0.57)	0.86 (0.77, 0.94)	0.83 (0.75, 0.91)
Eastern Sub‐Saharan Africa	477 (402–557)	0.78 (0.68–0.89)	6417 (3959–7954)	8.78 (5.12–11.06)	266 (153–335)	0.48 (0.25–0.62)	1205 (1030–1408)	0.87 (0.76–1.00)	18836 (11106–23606)	11.66 (6.54–14.69)	814 (450–1029)	0.66 (0.34–0.84)	0.47 (0.40, 0.55)	1.12 (1.06, 1.18)	1.23 (1.17, 1.29)
Non‐melanoma skin cancer (squamous‐cell carcinoma)															
Region															
Southern Sub‐Saharan Africa	1103 (895–1337)	4.21 (3.44–5.05)	3365 (2843–3755)	12.13 (10.12–13.60)	151 (125–170)	0.65 (0.54–0.74)	3514 (2854–4246)	6.51 (5.29–7.82)	8692 (7839–9435)	15.72 (14.19–17.00)	401 (362–433)	0.86 (0.77–0.93)	1.25 (0.62, 1.89)	1.21 (1.09, 1.32)	1.23 (1.13, 1.33)
Western Sub‐Saharan Africa	335 (284–392)	0.42 (0.37–0.49)	5268 (4410–6211)	5.64 (4.68–6.64)	205 (169–240)	0.27 (0.22–0.32)	810 (688–942)	0.48 (0.42–0.56)	16980 (12876–20755)	7.71 (5.90–9.26)	612 (471–732)	0.38 (0.29–0.44)	0.56 (0.52, 0.59)	1.30 (1.18, 1.41)	1.33 (1.22, 1.45)
Non‐melanoma skin cancer (basal‐cell carcinoma)															
Global															
Both	1194817 (1003244–1414118)	31.82 (26.70–37.62)	562 (257–1040)	0.01 (0.01–0.03)	—	—	3951466 (3488307–4463602)	48.80 (43.11–55.01)	1703 (782–3191)	0.02 (0.01–0.04)	—	—	2.23 (1.75, 2.72)	1.89 (1.46, 2.33)	—
Male	603786 (503497–720199)	37.34 (31.35–44.48)	274 (126–516)	0.02 (0.01–0.03)	—	—	2238819 (1971821–2534569)	61.42 (54.30–69.59)	933 (431–1739)	0.03 (0.01–0.05)	—	—	2.6 (2.04, 3.16)	2.25 (1.75, 2.75)	—
Female	591031 (495555–693623)	28.37 (23.81–33.24)	288 (131–537)	0.01 (0.01–0.03)	—	—	1712647 (1509698–1929286)	39.31 (34.70–44.26)	770 (357–1443)	0.02 (0.01–0.03)	—	—	1.75 (1.32, 2.18)	1.44 (1.06, 1.82)	—
Socio‐demographic Index															
High	869169 (725323–1042112)	84.49 (70.68–100.13)	385 (178–707)	0.04 (0.02–0.07)	—	—	3092344 (2747219–3465310)	172.17 (153.37–191.24)	1250 (583–2320)	0.07 (0.03–0.13)	—	—	3.48 (2.88, 2.09)	3.14 (2.58, 3.7)	—
High‐middle	203645 (175779–234580)	19.56 (16.97–22.49)	110 (51–208)	0.01 (0.00–0.02)	—	—	444878 (374730–520353)	22.06 (18.64–25.71)	233 (107–450)	0.01 (0.01–0.02)	—	—	0.43 (0.33, 0.54)	0.37 (0.28, 0.46)	—
Non‐melanoma skin cancer (basal‐cell carcinoma)															
Socio‐demographic Index															
Middle	81030 (67540–95851)	7.97 (6.64–9.36)	46 (21–88)	0.00 (0.00–0.01)	—	—	282680 (233627–331957)	11.38 (9.48–13.33)	159 (71–302)	0.01 (0.00–0.01)	—	—	1.44 (1.23, 1.64)	1.24 (1.05, 1.43)	—
Low‐middle	32233 (26643–38697)	5.60 (4.64–6.63)	17 (8–32)	0.00 (0.00–0.01)	—	—	97519 (78989–117419)	7.11 (5.79–8.47)	50 (22–95)	0.00 (0.00–0.01)	—	—	0.81 (0.70, 0.93)	0.75 (0.64, 0.86)	—
Low	8469 (6574–10699)	3.37 (2.66–4.15)	4 (2–9)	0.00 (0.00–0.00)	—	—	19022 (14945–24120)	3.37 (2.67–4.15)	10 (5–20)	0.00 (0.00–0.00)	—	—	0.21 (0.08, 0.33)	0.13 (0.02, 0.24)	—
Region															
Asia Pacific, high income	5305 (4314–6415)	2.74 (2.25–3.29)	4 (2–8)	0.00 (0.00–0.00)	—	—	17425 (14628–20804)	3.95 (3.30–4.73)	14 (6–27)	0.00 (0.00–0.01)	—	—	0.91 (0.80, 1.02)	0.76 (0.65, 0.86)	—
Central Asia	10413 (8272–12612)	22.65 (18.07–27.23)	6 (2–11)	0.01 (0.01–0.02)	—	—	16635 (12955–20530)	22.82 (18.02–27.70)	9 (4–17)	0.01 (0.01–0.02)	—	—	0.03 (0.02, 0.04)	0.0 (−0.01, 0.01)	—
East Asia	34453 (28170–41956)	3.78 (3.10–4.53)	23 (10–44)	0.00 (0.00–0.00)	—	—	246958 (206646–285908)	11.78 (9.97–13.55)	142 (63–275)	0.01 (0.00–0.01)	—	—	3.94 (3.4, 4.48)	3.52 (3.05, 3.99)	—
South Asia	8417 (6254–11419)	1.29 (0.97–1.66)	5 (2–9)	0.00 (0.00–0.00)	—	—	21286 (16355–27557)	1.42 (1.10–1.79)	12 (2–23)	0.00 (0.00–0.00)	—	—	0.36 (0.30, 0.41)	0.38 (0.33, 0.43)	—
Southeast Asia	5830 (4928–6795)	2.27 (1.94–2.63)	4 (2–7)	0.00 (0.00–0.00)	—	—	9981 (7485–12794)	1.63 (1.25–2.05)	6 (3–13)	0.00 (0.00–0.00)	—	—	−0.69 (−0.98, −0.40)	−0.65 (−0.91, −0.39)	—
Non‐melanoma skin cancer (basal‐cell carcinoma)															
Region															
Australasia	6725 (5294–8159)	29.25 (23.23–35.33)	4 (2–8)	0.02 (0.01–0.03)	—	—	13722 (10977–16820)	29.45 (23.54–35.74)	8 (4–16)	0.02 (0.01–0.03)	—	—	0.15 (0.04, 0.25)	0.10 (0.03, 0.18)	—
Caribbean	1960 (1573–2377)	7.44 (5.96–8.99)	1 (1–2)	0.00 (0.00–0.01)	—	—	3250 (2535–3980)	6.28 (4.91–7.70)	2 (1–4)	0.00 (0.00–0.01)	—	—	−0.58 (−0.69, −0.47)	−0.57 (−0.66, −0.47)	—
Central Europe	33348 (29301–37879)	23.09 (20.47–26.05)	18 (8–35)	0.01 (0.01–0.02)	—	—	62142 (51628–73676)	29.45 (24.72–34.49)	32 (15–61)	0.02 (0.01–0.03)	—	—	1.01 (0.87, 1.15)	0.83 (0.72, 0.94)	—
Eastern Europe	44224 (35328–53344)	16.17 (12.99–19.30)	24 (11–47)	0.01 (0.00–0.02)	—	—	65244 (51565–79858)	19.21 (15.24–23.24)	35 (16–68)	0.01 (0.00–0.02)	—	—	0.54 (0.48, 0.60)	0.45 (0.39, 0.51)	—
Western Europe	205656 (182848–232562)	36.60 (32.62–40.90)	115 (54–213)	0.02 (0.01–0.04)	—	—	315155 (255014–382285)	36.62 (29.67–44.13)	173 (80–327)	0.02 (0.01–0.04)	—	—	0.35 (0.17, 0.53)	0.29 (0.15, 0.44)	—
Andean Latin America	3493 (3142–3867)	17.39 (15.66–19.30)	2 (1–4)	0.01 (0.00–0.02)	—	—	8723 (6957–10631)	15.63 (12.53–19.11)	5 (2–10)	0.01 (0.00–0.02)	—	—	−0.21 (−0.52, 0.11)	−0.14 (−0.39, 0.11)	—
Central Latin America	24699 (20128–29561)	29.25 (23.89–35.01)	14 (6–26)	0.02 (0.01–0.03)	—	—	68781 (55333–82361)	29.16 (23.54–35.01)	37 (17–72)	0.02 (0.01–0.03)	—	—	0.01 (0.00, 0.01)	−0.11 (−0.02, 0.00)	—
Southern Latin America	10458 (9027–11991)	22.97 (19.85–26.33)	6 (3–12)	0.01 (0.01–0.03)	—	—	18645 (14878–22684)	22.61 (18.02–27.38)	11 (5–21)	0.01 (0.01–0.02)	—	—	−0.19 (−0.30, −0.08)	−0.18 (−0.27, −0.08)	—
Non‐melanoma skin cancer (basal‐cell carcinoma)															
Region															
Tropical Latin America	62198 (54790–70592)	67.11 (59.09–76.34)	26 (12–50)	0.03 (0.01–0.05)	—	—	178574 (155353–204151)	73.40 (63.75–83.86)	75 (35–143)	0.03 (0.01–0.06)	—	—	0.47 (0.33, 0.60)	0.52 (0.39, 0.65)	—
North Africa and Middle East	12758 (10657–15003)	7.43 (6.23–8.67)	8 (4–15)	0.00 (0.00–0.00)	—	—	29927 (23994–36217)	6.83 (5.50–8.26)	19 (8–36)	0.00 (0.00–0.01)	—	—	−0.02 (−0.31, 0.26)	−0.04 (−0.28, 0.21)	—
North America, high income	713279 (585827–865410)	253.90 (208.54–308.06)	296 (137–541)	0.00 (0.00–0.01)	—	—	2848027 (2537042–3178992)	781.22 (695.92–872.01)	1109 (518–2068)	0.00 (0.00–0.01)	—	—	4.07 (3.32, 4.82)	3.86 (3.12, 4.60)	—
Oceania	6 (3–10)	0.12 (0.06–0.20)	0 (0–0)	0.00 (0.00–0.00)	—	—	14 (6–23)	0.12 (0.06–0.19)	0 (0–0)	0.00 (0.00–0.00)	—	—	−0.01 (−0.01, 0.00)	−0.11 (−0.12, −0.11)	—
Central Sub‐Saharan Africa	1093 (826–1400)	4.57 (3.53–5.68)	1 (0–1)	0.00 (0.00–0.00)	—	—	2635 (1981–3411)	4.50 (3.44–5.59)	1 (1–3)	0.00 (0.00–0.00)	—	—	−0.05 (−0.07, −0.03)	−0.08 (−0.10, −0.06)	—
Eastern Sub‐Saharan Africa	3122 (2414–3962)	3.85 (3.02–4.76)	2 (1–3)	0.00 (0.00–0.00)	—	—	6876 (5303–8777)	3.76 (2.94–4.64)	4 (2–7)	0.00 (0.00–0.00)	—	—	−0.10 (−0.13, −0.08)	−0.12 (−0.14, −0.09)	—
Southern Sub‐Saharan Africa	4820 (3847–5885)	16.72 (13.24–20.42)	2 (1–5)	0.01 (0.00–0.02)	—	—	11702 (9253–14384)	19.85 (15.60–24.23)	6 (3–11)	0.01 (0.00–0.02)	—	—	0.17 (−0.43, 0.77)	0.15 (−0.37, 0.68)	—
Western Sub‐Saharan Africa	2560 (1937–3333)	2.66 (2.03–3.33)	1 (1–3)	0.00 (0.00–0.00)	—	—	5766 (4330–7588)	2.67 (2.04–3.35)	3 (1–6)	0.00 (0.00–0.00)	—	—	0.01 (−0.02, 0.04)	−0.02 (−0.05, 0.02)	—

Data from a total of 204 countries and territories were available. Geographically, the world was classified as 21 regions, on the basis of geographical proximity, sociocultural, and epidemiological similarities. The Socio‐demographic Index (SDI) is a summary measure of development status strongly correlated with health outcomes. According to the SDI 204 countries and territories were separated into 5 regions with low, low‐middle, middle, high‐middle, and high SDI.

Abbreviations: ASDR, age‐standardized disability‐adjusted life years (DALYs) rate; ASIR, age‐standardized incidence rate; ASMR, age‐standardized mortality rate; ASR, age standardized rate (per 100,000 population); CI, confidence interval; EAPC, estimated annual percentage change; SDI, socio‐demographic index; UI, uncertainty interval.

### 3.2 Regional‐ and national‐level incidence, mortality and burden of skin cancers in 2019

The numbers and corresponding ASR of incidence, DALYs and deaths of MSM were highest in high SDI regions in 2019. For SCC, the highest number of incident cases and ASIR also occurred in high SDI regions, while the greatest numbers and corresponding ASR of DALYs and deaths were in middle SDI regions. The highest numbers, ASIR and ASDR of BCC were observed in high SDI regions (Table 1). Geographically, the ASR of skin cancers varied markedly in 2019 (Table 1). The highest ASR of MSM was all found in Australasia. Regarding NMSCs, the largest incident number and ASIR of SCC were found to be in High‐income North America, and the highest numbers of DALYs and deaths were in East Asia, while Southern Sub‐Saharan Africa showed the greatest ASDR and ASMR. The greatest ASIR and ASDR of BCC were both observed in High‐income North America.

**FIGURE 2:**
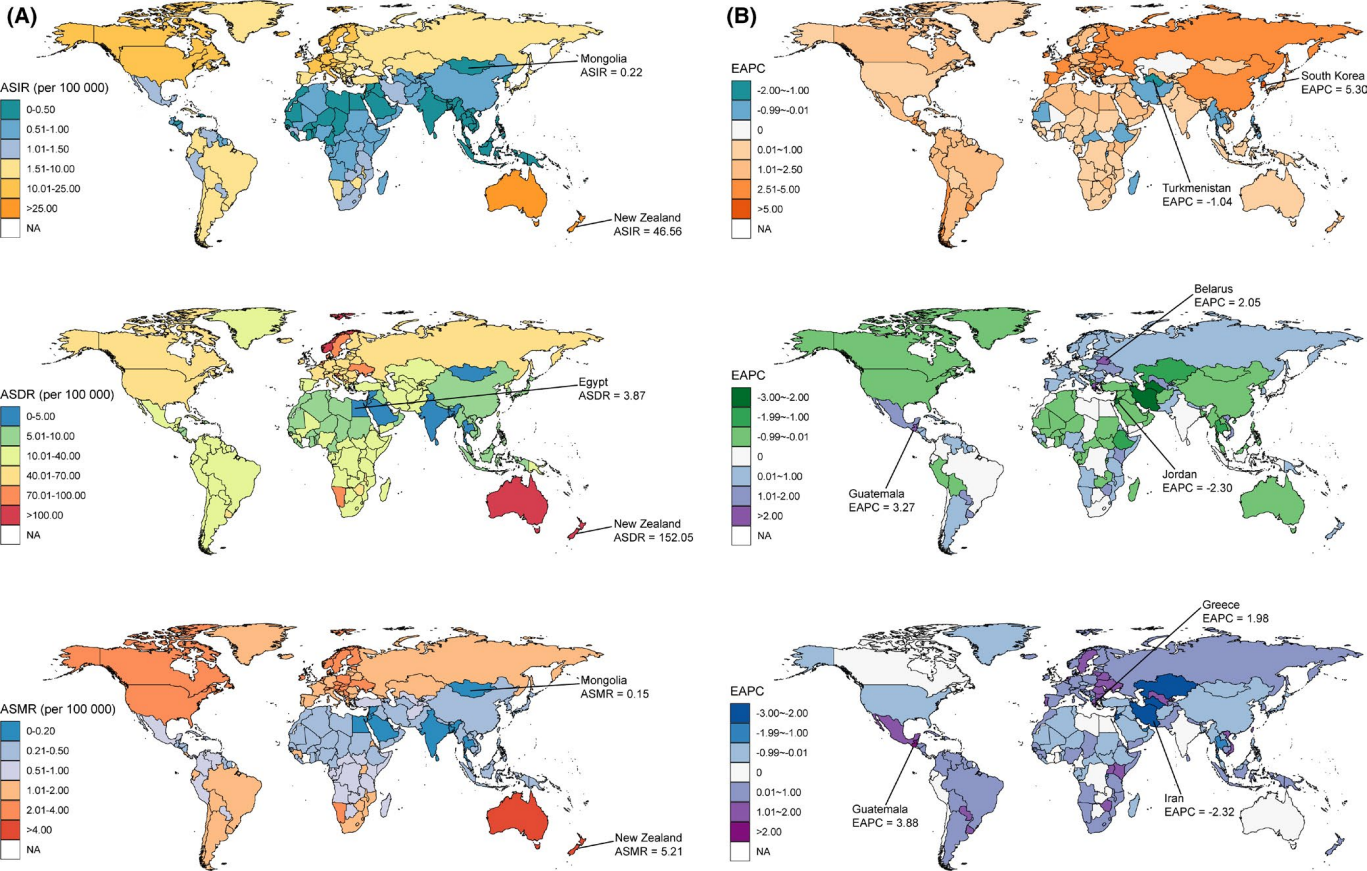
The global distribution of age‐standardized rates (ASR) of malignant skin melanoma incidence, disability‐adjusted life years (DALYs), and mortality for both sexes in 2019 (A, the left column), and the corresponding EAPCs of ASR from 1990 to 2019 (B, the right column). Abbreviations: ASIR, age‐standardized incidence rate; ASDR, age‐standardized DALY rate; ASMR, age‐standardized mortality rate

**FIGURE 3:**
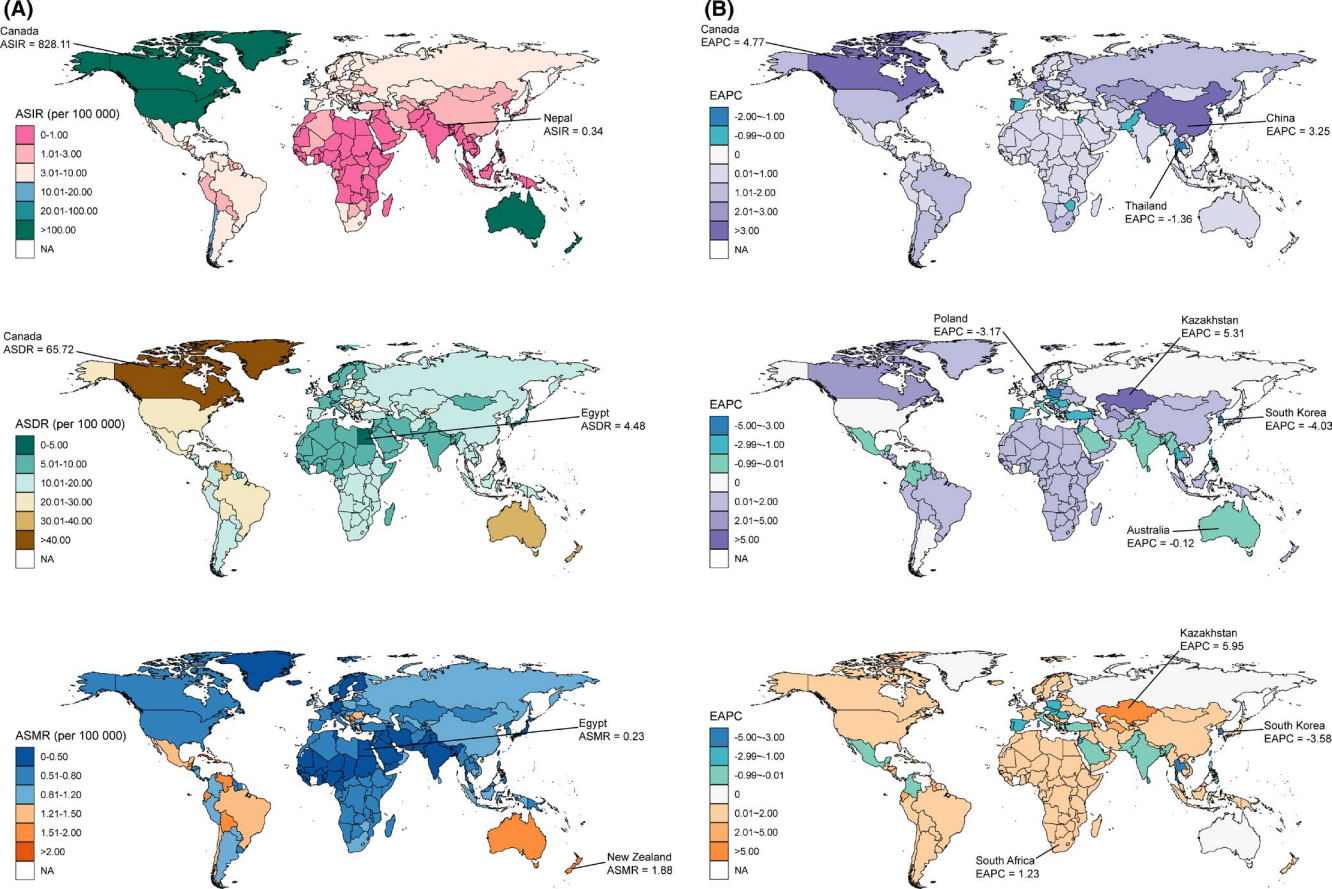
The global distribution of age‐standardized rates (ASR) of squamous‐cell carcinoma incidence, disability‐adjusted life years (DALYs), and mortality for both sexes in 2019 (A, the left column), and the corresponding EAPCs of ASR from 1990 to 2019 (B, the right column). Abbreviations: ASIR, age‐standardized incidence rate; ASDR, age‐standardized DALY rate; ASMR, age‐standardized mortality rate

**FIGURE 4:**
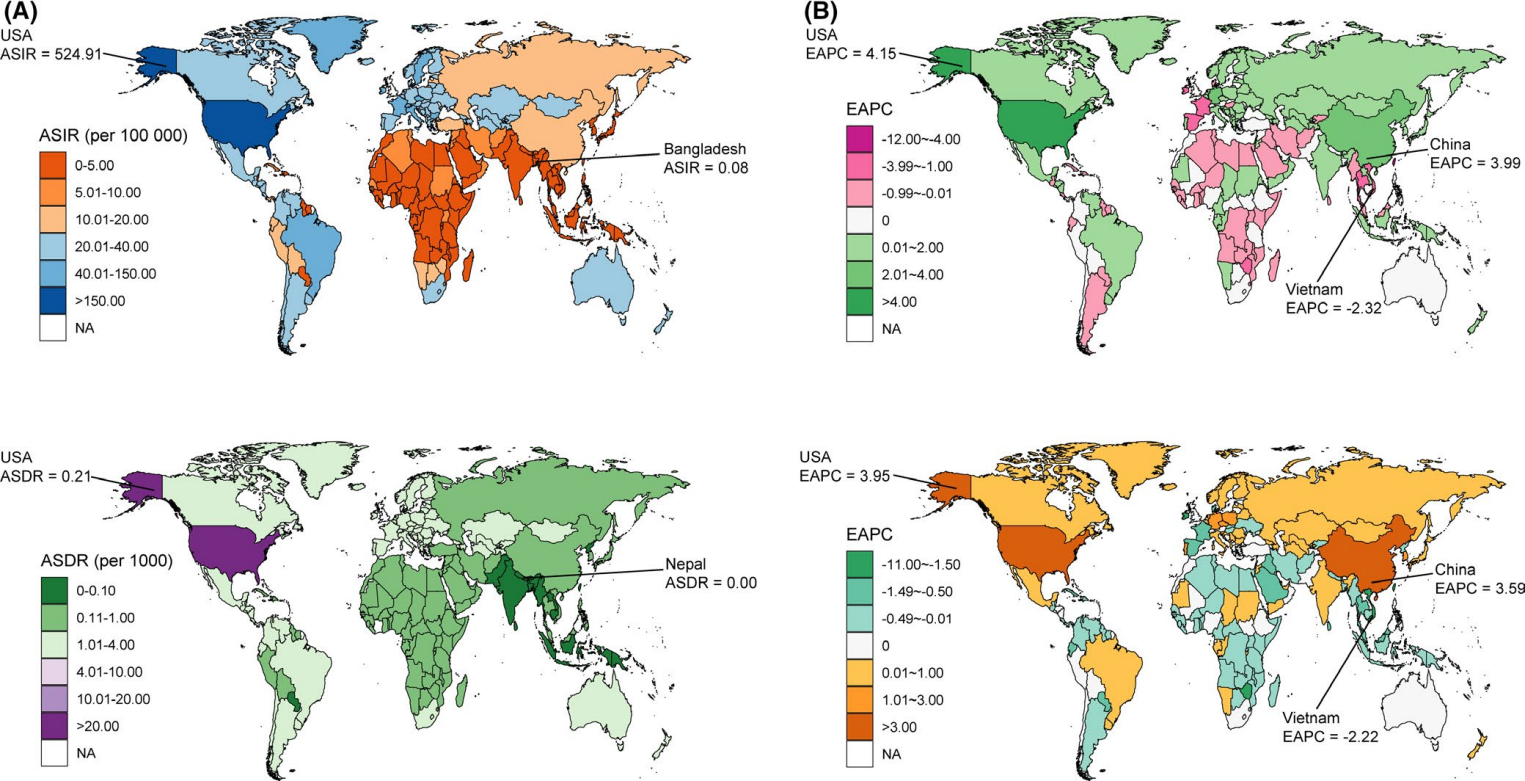
The global distribution of age‐standardized rates (ASR) of basal‐cell carcinoma incidence, disability‐adjusted life years (DALYs), and mortality for both sexes in 2019 (A, the left column), and the corresponding EAPCs of ASR from 1990 to 2019 (B, the right column). Abbreviations: ASIR, age‐standardized incidence rate; ASDR, age‐standardized DALY rate; ASMR, age‐standardized mortality rate

Figure 2A, Figure 3A and Figure 4A showed the variations in ASIR, ASDR, and ASMR of three skin cancers in 204 countries and territories in 2019. For instance, the ASIR of MSM ranged from 0.22 per 100,000 (95% UI: 0.14 to 0.32) in Sri Lanka to 46.56 per 100,000 (95% UI: 28.32 to 58.12) in New Zealand. The highest and lowest ASDR and ASMR of MSM were found in New Zealand (152.05 [95% UI: 101.46 to 179.36] and 5.21 [95% UI: 3.29 to 5.96]) and Mongolia (4.04 [95% UI: 2.96 to 5.95] and 0.15 [95% UI: 0.11 to 0.21]), respectively, and varied by a factor of more than 30 across countries.

### 3.3 Trends of the three skin cancers burden from 1990 to 2019

The ASIR of the three skin cancers significantly increased in most of the SDI and 21 geographical regions, with the largest increase observed in East Asia for MSM and SCC, and high‐income North America for BCC (Table 1, Figure 2B, Figure 3B, Figure 4B). The changes in ASDR and ASMR of the three skin cancers were heterogeneous across the regions. For MSM, the highest increase in ASDR and ASMR was observed in Eastern Europe and Central Latin America, respectively. For SCC, the greatest increase was observed in Central Asia, next was East Asia and Sub‐Saharan Africa. The ASDR of BCC in high‐income North America and East Asia showed the most pronounced increase.

There were considerable variations in the changes in ASR of the three skin cancers from 1990 to 2019 at the national level (Figure 2B, Figure 3B, Figure 4B). The ASIR of MSM was significantly increased in 167 countries and territories with the greatest one in South Korea (EAPC: 5.87, 95% CI: 5.40 to 6.34). While the ASIR of SCC also had a change in all 204 countries and territories (184 of which were increased), with the EAPC ranging from −1.36 (95% CI: −1.55 to −1.18) in Thailand to 4.77 (95% CI: 4.60 to 4.93) in Canada. Similar increased trends were found in most (110/204) of the countries and territories in terms of the ASIR of BCC, and the largest increase was found in USA (EAPC: 4.15, 95% CI: 3.39 to 4.92). The ASDR of MSM and BCC showed a significant decrease in about half of the countries and territories with the great decreases in Jordan (EAPC: −2.30, 95% CI: −2.60 to −2.01) and in Taiwan (China) (EAPC: −10.72, 95% CI: −11.83 to −9.60). Conversely, the ASDR of SCC significantly increased in 148 countries and territories with the largest EAPC in Bosnia and Herzegovina (7.04, 95% CI: 6.20 to 7.89). The ASMR of MSM significantly increased in 114 countries and territories. For SCC, 203 countries and territories showed changes in ASMR, 158 of which were increased, with the most pronounced increase in Bosnia and Herzegovina (EAPC: 8.28, 95% CI: 7.30 to 9.27).

## 4 DISCUSSION

In this study, we comprehensively analyzed the spatial and temporal trends in incidence, mortality and DALYs of skin cancers at the global, regional, and national level, by age, sex and subtype from 1990 to 2019. Although in some regions and countries, the primary and secondary prevention for BCC, such as UVR protection and early self‐detection, have the potential to substantially reduce morbidity and health care costs of BCC in recent years,^8^, ^10^, ^24^, ^25^ the global burden of BCC continues to grow in ASIR and ASDR. The global aging of the population has become more serious, which is probably the main reason for an increase in ASIR of BCC worldwide.^12^ Moreover, an unexpected and significant increase of ASIR due to BCC was observed in East Asia, in which the efforts for BCC prevention and screening programs should be made to reduce the healthcare costs and morbidity.^26^ In contrast to other regions in the world, the SCC mortality and DALYs in Southern Sub‐Saharan Africa in 2019, however, remained disproportionately high in comparison with incidence. Many patients with SCC and other cancers in sub‐Saharan Africa, presented with late‐stage disease, are thought to be associated with limited resources, lower socio‐economic status, poor disease awareness, difficulty detecting pigmented lesions in darker skin, little to no access to early detection and/or timely treatment,^27^, ^28^ a high burden of immunosuppression due to the HIV/AIDS epidemic in sub‐Saharan Africa and other sociocultural factors.^29^, ^30^, ^31^

Globally, in both sexes, there was a gradual decline in ASMR and ASDR of MSM from 1990 to 2019. The significant decrease in mortality and DALYs of MSM between 1990 and 2019 may be due in part to preventive measures such as self‐examination, public education, and development of new multiple treatment options in past decades, especially in some developed countries with high prevalence and incidence.^8^, ^10^, ^25^ However, there are still high incidence and great age‐standardized DALYs burdens caused by skin cancers in the high SDI regions or those developed countries, such as America, Australasia and Norway, in which the light‐skinned population accounts for a large proportion of the national population. The association of skin cancer with skin color is a significant etiologic factor except for UVR exposure.^2^, ^6^, ^32^ Skin cancer is less common in darkly pigmented people than in light‐skinned Caucasians, the former comes with more epidermal melanin to protect against UVR damage in human skin.^2^, ^28^ Moreover, we observed geographic variations in the incidence of skin cancers via the global heat map, with a higher incidence trends far away from the equator. Previous studies demonstrated that there was a significant association noted between human skin pigmentation and absolute latitude with darker pigmentation seen at the equator, and lighter skin seen closer to high latitudes,^33^, ^34^ which enable us to better understand the etiology of this geographic variability.

The EAPC provides a summary measure of the age‐standardized rate trend at a specified interval.^19^ Globally, the EAPC of BCC incidence in ASR increased, and, there is a significant increase in East Asia and Tropical Latin America which had been usually considered as low incidence areas. The EAPC of ASMR of SCC in both sexes is high from 1990 to 2019 was Central Asia, East Asia, and Sub‐Saharan Africa. Strikingly, ASR trends were showed a high contribution to incidence, DALYs and/or mortality in East Asia, Sub‐Saharan Africa and Tropical Latin America, in which was assumed there was a low disease burden of skin cancers in the past. It may be due to the high ASIR, ASDR and ASMR coupled with the expanding population in these large and populous countries such as China and Brazil. Africa also has high rate of population growth among major areas, which is expected to reach more than fifty percent of the global population increase by 2050.^35^ The other potential reason is that the rising incidence of age‐related skin cancer has presented in these regions under the background an aging global population. Further investigations are required to better understand the reasons for the continuous growth of skin cancer burdens in these regions. Altogether, despite the temporal trends of DALYs and death due to MSM globally declined over the past 29 years, especially in High‐income North America region, which benefited from primary and secondary prevention, and new multiple treatment development in past decades, there were undesirable increases in many regions particularly in less developed regions with rapid population growth, and importantly, more attention should be paid to skin cancer control among these regions and countries. In terms of SCC and BCC there has been consistent growth on the skin burdens worldwide, therefore increased efforts are also needed in SCC and BCC prevention. In addition, a previous study, conducted by Urban et al, assessed the global trends in the skin cancer in 195 countries from 1990 to 2017 worldwide via the GBD 2017study, which mainly highlighted the trend of prevalence and DALYs posed by skin cancer.^36^ Thus, our study might provide the significant extension and complement for the previous study.

Some limitations of the GBD 2019 assessment on skin cancer should be noted. The GBD analysis of relevant skin cancer data on histology subtypes, risk factors, and BCC mortality is not covered. Therefore, the spatial and temporal trends in incidence, DALYs and death of skin cancer stratified by these factors were not estimated in this study. Additionally, another crucial deficit is the quality of GBD skin cancer data, such as underreporting of skin cancer, the heterogeneity of data sources and definitions of skin cancers.

In summary, the results of our study highlight the high worldwide burden of skin cancer variations by country and region, which indicated that skin cancer remains a significant public health concern globally. Australasia, high‐income North America and Europe in particular, will continue to be the areas most burdened by skin cancers. The incidence and/or mortality rates of skin cancers showed a significant increase trend in East Asia, Latin America, and Sub‐Saharan Africa due in part to their increase in population density and inadequate preventive and control measures. Current strategies for reducing the incidence and mortality of skin cancer should be re‐evaluated, and the focus should be on preventive measures, such as public education, UVR protection, self‐examination, and screening programs, especially in the countries and regions with a high or increasing incidence rate and/or mortality rate, especially for the population over 55 years old.

## PATIENT AND PUBLIC INVOLVEMENT

Patients or the public were not involved in the design, data collection, analyses, or interpretation of this research.

## CONFLICT OF INTEREST

The authors declare to no conflicts of interest that pertain to this work.

## ETHICS APPROVAL

This study was approved by Ethics committee of Affiliated Hospital of Guizhou Medical University, Guiyang 550001, Guizho, China (Approval No.2020151).

## Data Availability

The data used to support the findings of this study were extracted from the GBD 2019 database, which is freely available on http://ghdx.healthdata.org/gbd‐results‐tool.

## References

[1] Lomas A , Leonardi‐Bee J , Bath‐Hextall F . A systematic review of worldwide incidence of nonmelanoma skin cancer. Br J Dermatol. 2012;166(5):1069‐1080. 22251204 10.1111/j.1365-2133.2012.10830.x

[2] Gordon R . Skin cancer: an overview of epidemiology and risk factors. Semin Oncol Nurs. 2013;29(3):160‐169. 23958214 10.1016/j.soncn.2013.06.002

[3] Cameron MC , Lee E , Hibler BP , et al. Basal cell carcinoma: epidemiology; pathophysiology; clinical and histological subtypes; and disease associations. J Am Acad Dermatol. 2019;80(2):303‐317. 29782900 10.1016/j.jaad.2018.03.060

[4] Guy GP Jr , Machlin SR , Ekwueme DU , et al. Prevalence and costs of skin cancer treatment in the U.S., 2002‐2006 and 2007‐2011. Am J Prev Med. 2015;48(2):183‐187. 25442229 10.1016/j.amepre.2014.08.036 PMC4603424

[5] Fitzmaurice C , Abate D , Abbasi N , et al. Global, regional, and national cancer incidence, mortality, years of life lost, years lived with disability, and disability‐adjusted life‐years for 29 cancer groups, 1990 to 2017: a systematic analysis for the global burden of disease study. JAMA Oncol. 2019;5(12):1749–1768. 31560378 10.1001/jamaoncol.2019.2996 PMC6777271

[6] Gloster HM Jr , Neal K . Skin cancer in skin of color. J Am Acad Dermatol. 2006;55(5):741‐760; quiz 761‐744. 17052479 10.1016/j.jaad.2005.08.063

[7] Moslehi R , Zeinomar N , Boscoe FP . Incidence of cutaneous malignant melanoma in Iranian provinces and American States matched on ultraviolet radiation exposure: an ecologic study. Environ Pollut. 2018;234:699‐706. 29241156 10.1016/j.envpol.2017.11.099 PMC5921862

[8] Brunssen A , Waldmann A , Eisemann N , et al. Impact of skin cancer screening and secondary prevention campaigns on skin cancer incidence and mortality: a systematic review. J Am Acad Dermatol. 2017;76(1):129‐139.e110. 27707591 10.1016/j.jaad.2016.07.045

[9] Simoes MCF , Sousa JJS , Pais A . Skin cancer and new treatment perspectives: a review. Cancer Lett. 2015;357(1):8‐42. 25444899 10.1016/j.canlet.2014.11.001

[10] Wernli KJ , Henrikson NB , Morrison CC , et al. Screening for skin cancer in adults: updated evidence report and systematic review for the us preventive services task force. JAMA. 2016;316(4):436‐447. 27458949 10.1001/jama.2016.5415

[11] Segal UA . Globalization, migration, and ethnicity. Public Health. 2019;172:135‐142. 31229257 10.1016/j.puhe.2019.04.011

[12] Beard JR , Officer A , de Carvalho IA , et al. The world report on ageing and health: a policy framework for healthy ageing. Lancet. 2016;387(10033):2145‐2154. 26520231 10.1016/S0140-6736(15)00516-4 PMC4848186

[13] Lucas RM , Yazar S , Young AR , et al. Human health in relation to exposure to solar ultraviolet radiation under changing stratospheric ozone and climate. Photochem Photobiol Sci. 2019;18(3):641‐680. 30810559 10.1039/c8pp90060d

[14] Rogers HW , Weinstock MA , Feldman SR , et al. Incidence estimate of nonmelanoma skin cancer (keratinocyte carcinomas) in the U.S. Population, 2012. JAMA Dermatol. 2015;151(10):1081‐1086. 25928283 10.1001/jamadermatol.2015.1187

[15] Lubeek SFK , van Vugt LJ , Aben KKH , et al. The epidemiology and clinicopathological features of basal cell carcinoma in patients 80 years and older: a systematic review. JAMA dermatol. 2017;153(1):71‐78. 27732698 10.1001/jamadermatol.2016.3628

[16] Seidl S , Schuster B , Rüth M , et al. What do Germans want to know about skin cancer? A nationwide google search analysis from 2013 to 2017. J Med Internet Res. 2018;20(5):e10327. 29698213 10.2196/10327 PMC5956155

[17] Perera E , Gnaneswaran N , Staines C , et al. Incidence and prevalence of non‐melanoma skin cancer in Australia: a systematic review. Australas J dermatol. 2015;56(4):258‐267. 25716064 10.1111/ajd.12282

[18] GBD 2017 Disease and Injury Incidence and Prevalence Collaborators . Global, regional, and national incidence, prevalence, and years lived with disability for 354 diseases and injuries for 195 countries and territories, 1990–2017: a systematic analysis for the global burden of disease study 2017. Lancet. 2018;92(10159):1789‐1858. 10.1016/S0140-6736(18)32279-7 PMC6227754 30496104

[19] Gao S , Yang WS , Bray F , et al. Declining rates of hepatocellular carcinoma in urban Shanghai: incidence trends in 1976–2005. Eur J Epidemiol. 2012;27(1):39‐46. 22160277 10.1007/s10654-011-9636-8 PMC5477645

[20] GBD 2017 Causes of Death Collaborator . Global, regional, and national age‐sex‐specific mortality for 282 causes of death in 195 countries and territories, 1980–2017: a systematic analysis for the global burden of disease study 2017. Lancet. 2018;392(10159):1736‐1788. 30496103 10.1016/S0140-6736(18)32203-7 PMC6227606

[21] GBD 2017 DALYs and HALE Collaborator . Global, regional, and national disability‐adjusted life‐years (DALYs) for 359 diseases and injuries and healthy life expectancy (HALE) for 195 countries and territories, 1990–2017: a systematic analysis for the global burden of disease study 2017. Lancet. 2018:392(10159):1859‐1922. 30415748 10.1016/S0140-6736(18)32335-3 PMC6252083

[22] Li X , Cao X , Guo M , Xie M , Liu X . Trends and risk factors of mortality and disability adjusted life years for chronic respiratory diseases from 1990 to 2017: systematic analysis for the Global Burden of Disease Study 2017. BMJ. 2020;m234. 10.1136/bmj.m234. 32075787 PMC7190065

[23] Liu Z , Jiang Y , Yuan H , et al. The trends in incidence of primary liver cancer caused by specific etiologies: results from the Global Burden of Disease study 2016 and implications for liver cancer prevention. J Hepatol. 2019;70(4):674‐683. 30543829 10.1016/j.jhep.2018.12.001

[24] Morgan FC , Duran J , Fraile B , et al. A comparison of skin cancer screening and treatment costs at a Massachusetts cancer center, 2008 versus 2013. J Am Acad Dermatol. 2018;79(5):921‐928. 30322559 10.1016/j.jaad.2018.06.045

[25] Ferris LK . The value of behavioral counseling for skin cancer prevention: actions we can take now and guidance for the future. JAMA oncology. 2018;4(5):630‐632. 29558534 10.1001/jamaoncol.2018.0469

[26] Gordon LG , Elliott TM , Wright CY , et al. Modelling the healthcare costs of skin cancer in South Africa. BMC Health Serv Res. 2016;16(1):113. 27039098 10.1186/s12913-016-1364-z PMC4818961

[27] Brinton LA , Figueroa JD , Awuah B , et al. Breast cancer in Sub‐Saharan Africa: opportunities for prevention. Breast Cancer Res Treat. 2014;144(3):467‐478. 24604092 10.1007/s10549-014-2868-z PMC4023680

[28] Nthumba PM , Cavadas PC , Landin L . Primary cutaneous malignancies in Sub‐Saharan Africa. Ann Plast Surg. 2011;66(3):313‐320. 21233701 10.1097/SAP.0b013e3181e7db9a

[29] Chinula L , Moses A , Gopal S . HIV‐associated malignancies in Sub‐Saharan Africa: progress, challenges, and opportunities. Curr Opin HIV and AIDS. 2017;12(1):89‐95. 27607593 10.1097/COH.0000000000000329 PMC5241291

[30] Halder RM , Ara CJ . Skin cancer and photoaging in ethnic skin. Dermatol Clin. 2003;21(4):725–7. 14717413 10.1016/s0733-8635(03)00085-8

[31] Wilkins K , Turner R , Dolev JC , et al. Cutaneous malignancy and human immunodeficiency virus disease. J Am Acad Dermatol. 2006;54(2):189‐206. quiz 207‐110. 16443048 10.1016/j.jaad.2004.11.060

[32] Kozma B , Photocarcinogenesis EMJ . An epidemiologic perspective on ultraviolet light and skin cancer. Dermatol Clin. 2014;32(3):301‐313. 24891053 10.1016/j.det.2014.03.004

[33] Martin AR , Lin M , Granka JM , et al. An unexpectedly complex architecture for skin pigmentation in Africans. Cell. 2017;171(6):1340‐1353.e14. 29195075 10.1016/j.cell.2017.11.015 PMC5884124

[34] Sturm RA , Duffy DL . Human pigmentation genes under environmental selection. Genome Biol. 2012;13(9):248. 23110848 10.1186/gb-2012-13-9-248 PMC3491390

[35] United Nations DoEaSA, Population Division: World Population Prospects: The 2015 Revision, Key Findings and Advance Tables . Working Paper No ESA/P/WP241. 2015.

[36] Urban K , Mehrmal S , Uppal P , et al. The global burden of skin cancer: a longitudinal analysis from the global burden of disease study, 1990–2017. JAAD International. 2021;2:98‐108. 10.1016/j.jdin.2020.10.013 PMC8362234 34409358

